# P05 *In vivo* efficacy of VRP-034 in lung and thigh infection models

**DOI:** 10.1093/jacamr/dlac004.004

**Published:** 2022-02-16

**Authors:** Kamlesh Kumar Vishwakarma, Anurag Payasi, Shailesh Kumar, Arun Sharma, Saransh Chaudhary, Anmol Aggarwal

**Affiliations:** Venus Medicine Research Centre, India; Venus Medicine Research Centre, India; Venus Medicine Research Centre, India; Venus Medicine Research Centre, India; Venus Medicine Research Centre, India; Venus Medicine Research Centre, India

## Abstract

**Background:**

Polymyxin B-induced kidney injury is an important clinical concern that undermines patient care. This injury occurs in 30%–60% of patients receiving systemic polymyxin B (PMB). VRP-034, a novel PMB formulation, has previously shown a promising safety profile as compared with marketed PMB in animal models (Roy *et al*.^1^). The objective of this study was to assess the efficacy of VRP-034 versus marketed PMB in murine lung and thigh infection models.

**Materials and methods:**

26 neutropenic BALB/c mice (*n = *12 for lung; *n = *14 for thigh) were infected by inoculating *Pseudomonas aeruginosa* (ATCC 27853, pathogenic, PMB MIC 0.5 μg/mL) either into the lungs (5 × 10^6^ cfu) via intratracheal administration (lung model) or in both thighs (10^6^ cfu/thigh) via intramuscular administration (thigh model). Treatment mice received VRP-034 or marketed PMB subcutaneously at 8 mg/kg every 8 h (HED: 2 mg/kg/day). Mice were humanely euthanized at 0 h (control) and at different timepoints post-treatment (24 h—lung model; 6 h and 12 h—thigh model). Lung or thigh tissues were collected, homogenized, serially diluted, plated on permissive media with cfu counted after 24 h of incubation. Changes in log_10_ cfu/mL at each timepoint were compared with 0 h control to assess efficacy.

**Results:**

In the lung infection model, the mean (±SEM) log_10_ cfu/mL increased from 8.01 ± 0.43 (at 0 h) to 12.02 ± 0.85 (at 24 h) in the vehicle control group. Treatment with marketed PMB and VRP-034 resulted in 1.96 ± 0.87 and 2.07 ± 0.42 log-reduction respectively in bacterial burden when compared with 0 h control. In the thigh infection model, the log_10_ cfu/mL increased from 6.40 ± 0.11 (at 0 h) to 9.85 ± 0.08 (at 6 h) and 11.23 ± 0.07 (at 12 h) in the vehicle control group. Treatment with marketed PMB reduced the bacterial burden by 0.65 ± 0.08 and 2.79 ± 0.14 log at 6 h and 12 h, respectively and treatment with VRP-034 reduced the bacterial burden by 0.85 ± 0.17 and 2.68 ± 0.01 log at 6 h and 12 h, respectively.

**Conclusions:**

The results from the murine lung and thigh infection models suggest that there is no marked difference between the efficacy of VRP-034 and marketed PMB. Further work should explore the clinical utility of these findings in the light of the promising safety profile of VRP-034.
